# Diagnosis of Carney complex following multiple recurrent cardiac myxomas

**DOI:** 10.1007/s11748-021-01719-w

**Published:** 2021-10-12

**Authors:** Shigeki Yokoyama, Kanetsugu Nagao, Akihiko Higashida, Masaya Aoki, Shigeyuki Yamashita, Nobuyuki Fukuda, Toshio Doi, Akio Yamashita, Kazuaki Fukahara, Naoki Yoshimura

**Affiliations:** 1 Department of Surgery 1, Faculty of Medicine, University of Toyama, 2630 Sugitani, Toyama, 930-0194 Japan; 2 Second Department of Internal Medicine, University of Toyama, Toyama, Japan

**Keywords:** Carney complex, Recurrent of cardiac myxoma, Cryoablation for myxoma

## Abstract

Carney complex is a rare syndrome caused by a genetic mutation leading to multiple endocrine abnormalities and a variety of tumors. Here, we report a case of Carney complex diagnosed due to recurrent multiple myxomas in the right atrium of a patient 16 years after the resection of the primary left atrial myxoma. Surgical excision was performed for the multiple recurrent right atrial tumors under cardiopulmonary bypass. The patient remained complication-free after surgery and was discharged on the 14th day. He was scheduled to continue echocardiographic follow-up and periodic systemic review by an endocrinologist. This case emphasizes the fact that if cardiac myxomas tend to be multiple and recurrent at a relatively young age, the possibility of Carney complex should be considered, even in the absence of any other related feature other than cardiac tumors.

## Introduction

Carney complex (CNC) is a rare syndrome of multiple endocrine neoplasia characterized by characteristic pigmented lesions on the skin and mucosal surfaces, cardiac and non-cardiac mucinous tumors, and multiple endocrine tumors [1, 2]. Fifty-three percent of CNC cases are reported to be associated with cardiac myxomas, and relapses are common (12–22%). Furthermore, 57% of deaths associated with CNC are cardiac-related deaths, including those due to cardiac tumors. Thus, early detection and treatment of cardiac myxoma-related complications are crucial in patients with CNC [3]. Here, we report a case of CNC diagnosed 16 years after left atrial myxoma resection, discovered due to recurrent multiple myxomas in the right atrium.

## Case

**Fig. 1:**
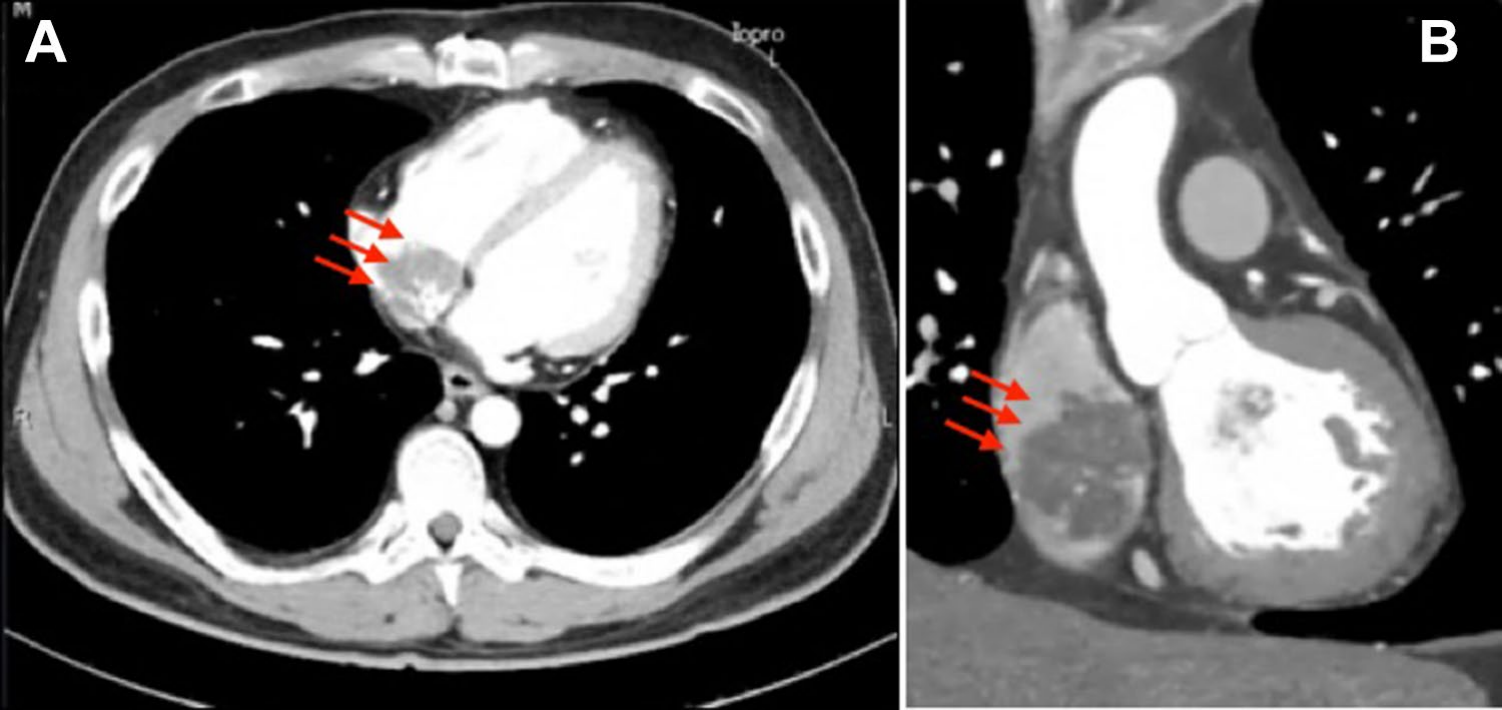
Preoperative computed tomography images showing a 32 × 26-mm-diameter tumor attached to the atrial septum (red arrow) **a** Axial view **b** Coronal view

**Fig. 2:**
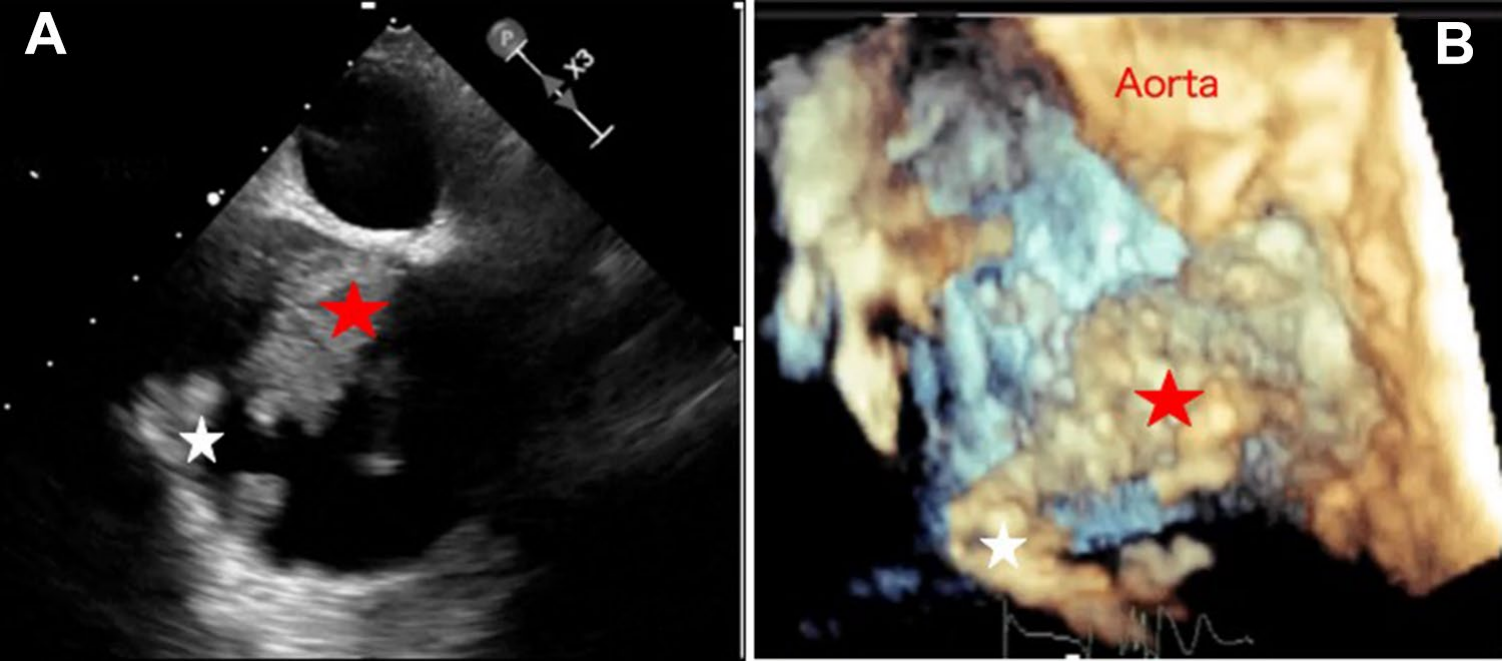
Transesophageal echocardiography (TEE) revealed a tumor adherent to the atrial septum (red star) as well as another 5-mm tumor on the anterior wall of the right atrium (white star) **a** Image of the right atrial tumors on TEE **b** Image of the right atrial tumors on three-dimensional TEE

**Fig. 3:**
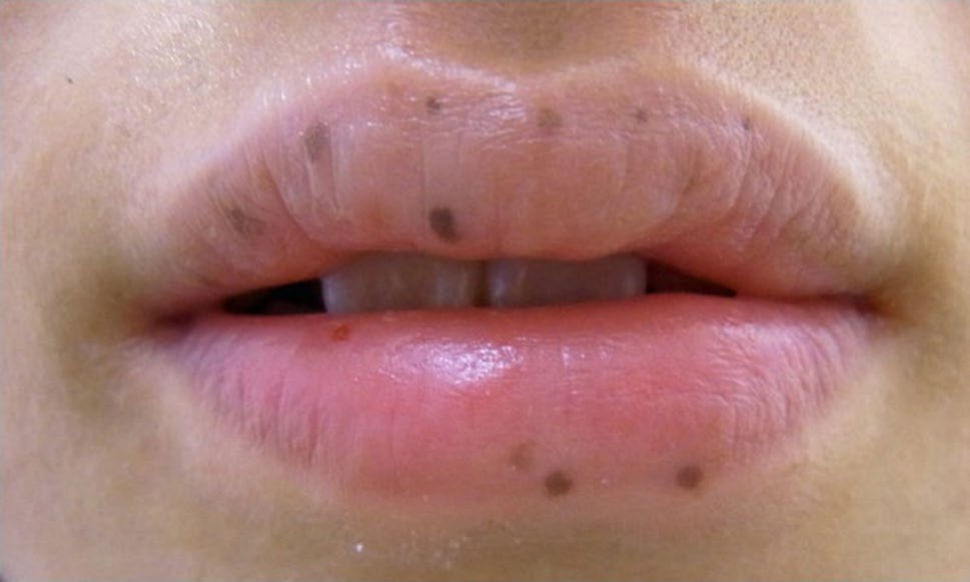
Multiple lip pigmentation

**Fig. 4:**
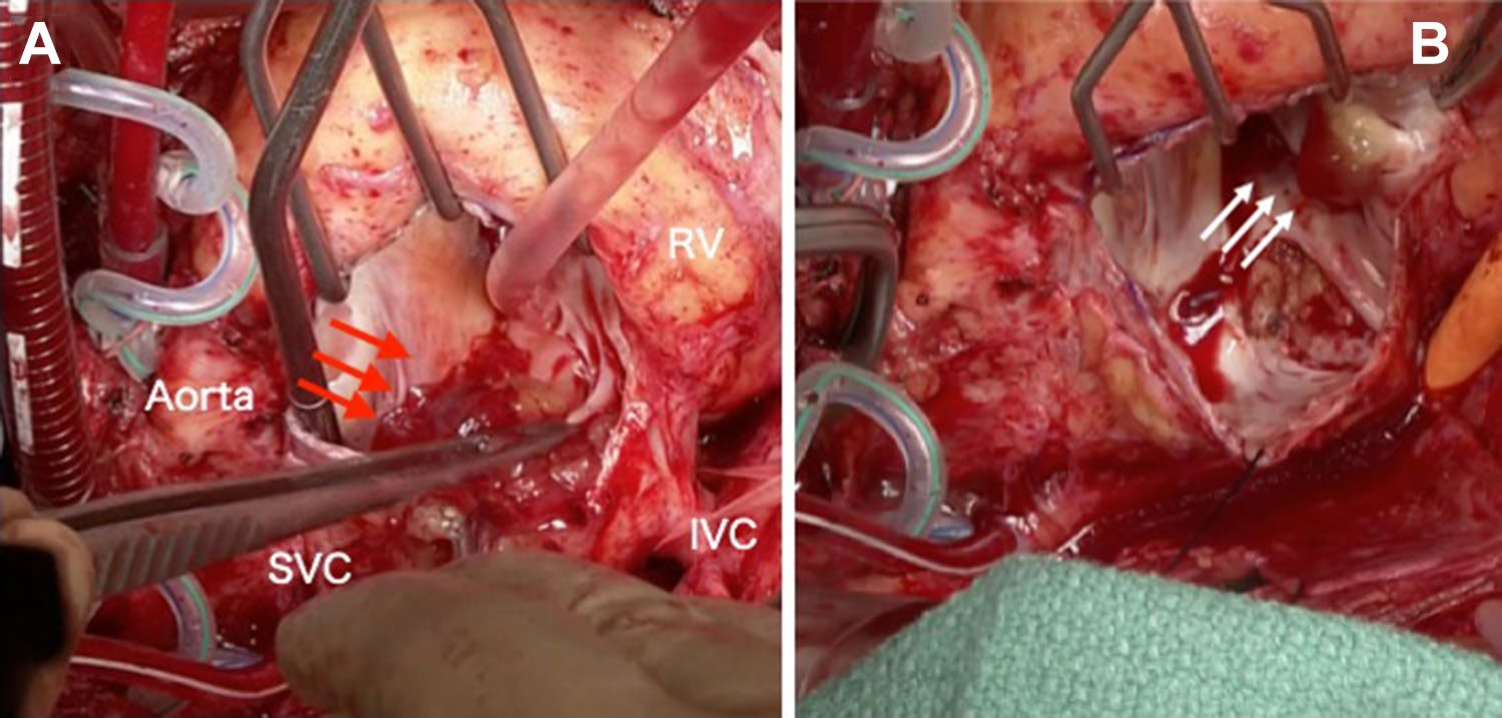
Intraoperative findings **a** Jelly-like tumor with a broad 15 × 10-mm stalk located in the coronary sinus near Koch's triangle and in the fossa ovalis (red arrow) **b** Second small tumor of the same shape with an approximately 5 × 5-mm stalk present in the free wall of the right atrium near the right coronary artery (white arrow). *RV* right ventricle, *SVC* superior vena cava, *IVC* inferior vena cava

**Fig. 5:**
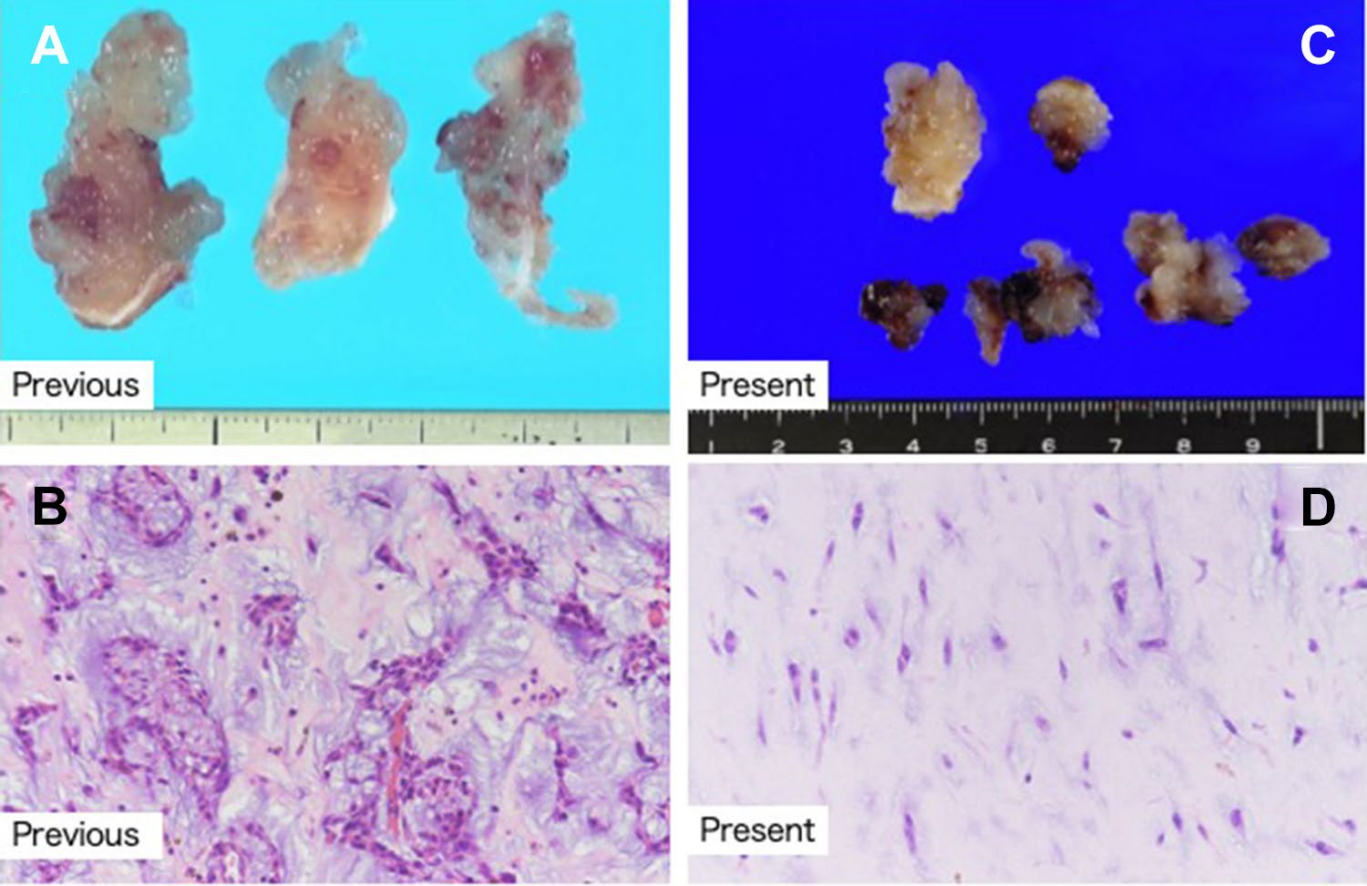
Pathological findings **a** Specimen of a left atrial myxoma removed 16 years ago. A papillomatous lesion with a grape-like appearance and another specimen of stalked growth from a fibrous tissue **b** Histopathological staining images of the specimen removed 16 years ago showing a scattered appearance of small spindle-shaped cells against a background of the myxoid interstitium **c** Specimen of an excised right atrium myxoma of the present case **d** Histopathological staining images of specimens of the present case showing a scattered appearance of small spindle-shaped cells against a background of the myxoid interstitium

A 41-year-old man presented to our hospital for surgery after a transthoracic echocardiogram conducted by his family doctor revealed a right atrial tumor. The patient had previously undergone a left atrial myxomectomy at the age of 25 years. He was diagnosed after a systemic examination due to stroke at a young age. Initial operative findings revealed a jelly-like tumor located in the left atrium measuring 25 × 15 mm with a broad stalk in the atrial septum. Due to young age and relatively large stalk, the atrial septum was extensively resected with margins and closed with expanded polytetrafluoroethylene (ePTFE) patch. Thereafter, the patient underwent regular postoperative echocardiographic examinations and had no evidence of intracardiac tumors until the age of 40 years. However, at the age of 41 years, the first postoperative neoplastic lesion in the right atrium was noted, and the patient was referred to our hospital. Physical examination performed following admission to the hospital revealed pigmented patches on the face and lips. His blood pressure and pulse rate were normal, and he had no subjective symptoms. Transthoracic echocardiography revealed a mobile tumor in the right atrium. In diastole, part of the tumor extended through the tricuspid valve into the right ventricle. Cardiac computed tomography showed that the tumor was attached to the atrial septum and was 32 × 26 mm in diameter (Fig. 1). Transesophageal echocardiography showed a tumor adherent to the atrial septum as well as another 5-mm large tumor on the anterior wall of the right atrium (Fig. 2). CNC was suspected owing to the presence of recurrent multiple cardiac tumors and the presence of pigmented spots on the lips (Fig. 3). After psychological support, including counseling, genetic testing was performed, which revealed a heterozygous p.Val164fs mutation in the coding region of the PRKAR1A gene, leading to the diagnosis of the Carney complex [4]. Surgical excision was planned for the multiple right atrial tumors. The surgery was performed through a midline sternal incision. After systemic heparinization, cardiopulmonary bypass was established with ascending aortic cannulation and venous drainage from the bicaval cannulation. The ascending aorta was cross-clamped, and cardiac arrest was achieved with a cardioplegic solution infusion from the aortic root. After the superior and inferior vena cavas were snared, the right atrium was opened. The tumor was jelly-like in texture with a broad 15 × 10-mm stalk located in the coronary sinus near Koch's triangle and in the fossa ovalis (Fig. 4a). Moreover, a small tumor of the same shape with an approximately 5 × 5-mm stalk was present in the free wall of the right atrium near the right coronary artery (Fig. 4b). The two tumors were resected, each with a part of Koch's triangle and right atrial endocardium close to the right coronary artery. Resection of wide tumor margins was not achievable as this would have led to resection of the entire atrial wall; thus, additional cryoablation was applied to the resection margins of the tumors. Each intimal defect was then closed directly using 5–0 prolene continuous sutures. The intracardiac cavity was thoroughly washed to ensure that there was no residual tumor. Weaning from the cardiopulmonary bypass was uneventful. The operative time was 240 min, and the cardiopulmonary and cardiac arrest durations were 95 and 51 min, respectively. The pericardium was closed with ePTFE sheets owing to the possibility of reoperation due to tumor recurrence in the future. Postoperative transthoracic echocardiography showed no residual tumor and normal tricuspid valve function. Pathological diagnosis of the resected tumor showed scattered small spindle-shaped cells against a background of myxoid stroma, leading to the diagnosis of myxoma (Fig. 5) [5]. The postoperative course was uneventful, with no atrioventricular block, and the patient was discharged on the 14th day. The patient was scheduled for permanent echocardiographic follow-up and systemic review by an endocrinologist.

## Discussion

Primary cardiac tumors are found in 0.0017%–0.19% of autopsied cases, and 75% of these are benign. Cardiac myxomas account for approximately 50% of primary benign cardiac tumors and are often solitary, with the left atrium being the most common site of origin [5]. CNC, described by Carney et al. in 1985, is a syndrome characterized by skin pigmentation, endocrine hyperplasia (including adrenocortical tumors and pituitary adenomas), and cardiac myxomas [1–4]. It has been reported that 5.9% of cardiac myxomas have CNC, and 53% are associated with cardiac tumors. The differences between solitary cardiac myxomas and those associated with CNC are that CNC-associated myxomas occur at an earlier age, are more frequent, show familial association due to autosomal dominant inheritance, and have a higher recurrence rate (12–22%). Of all CNC-related deaths, 57% are due to cardiac tumors. In the present case, CNC was suspected due to the young age of the patient (25 years) at the time of diagnosis of the first cardiac myxoma, recurrent multiple cardiac myxomas, and pigmented spots on the lips. [3]. No evidence of CNC in his relatives was noted, and he was considered to have isolated CNC. Cardiovascular surgeons need to be knowledgeable about CNC, as they may be the first to identify a CNC following a cardiac myxoma, as was evident in this case [6, 7]. CNC is caused due to a genetic mutation that can cause multiple endocrine abnormalities and a variety of tumors, and we need to be aware of this and detect abnormalities early. In patients diagnosed with cardiac myxomas, the following protocol is recommended: (1) cardiac ultrasound (once a year in adult patients with previous myxoma resection), (2) testicular ultrasound (once a year), (3) thyroid ultrasound (once a year), (4) intra-abdominal ultrasound of the ovaries (once a year), (5) urinary free cortisol measurement (once a year), and (6) serum IGF-1 level measurement (once a year) [6, 7].

The cause of recurrent cardiac myxedema in CNC may be genetic in origin. They can occur in the atria as well as in the ventricular muscles, making complete surgical prevention difficult. However, other reasons for recurrence may include: (1) incomplete resection of the tumor or (2) residual pre-tumor of some kind in the vicinity of the tumor [8]. Therefore, in the case of CNC of atrial septal origin, atrial septal resection is preferable whenever possible. In the present case, the atrial septum was extensively resected at the initial surgery and there was almost no remaining atrial septum that could be resected; therefore, no additional prophylactic resection could be performed. On the other hand, we decided to add cryocoagulation to eliminate the concern of a kind of pre-tumor remnant in the vicinity of the tumor. Cryoablation for cardiac myxomas has been used in patients with recurrent disease and reported to have good short-term results [9, 10]. In this case, to preserve the important anatomical structures, cryoablation was applied to the resection margins of the tumors. We believe that recurrence of myxoma in the heart is possible, especially since this was a CNC case, and thus, cryoablation was useful in maintaining the intracardiac structures. The pericardium was closed with ePTFE sheets owing to the possibility of reoperation due to tumor recurrence in the future. The patient did not suffer from atrioventricular block, and postoperative echocardiography showed that cardiac function was maintained.

## Conclusion

We have reported a case of CNC diagnosed 16 years after left atrial myxoma surgery, discovered due to multiple recurrent right atrial myxomas. It can be concluded that in a young patient with recurrent cardiac myxomas, the possibility of CNC must be considered even in the absence of other related features. Thorough evaluation of family history coupled with genetic diagnosis and appropriate consultation with an endocrinologist are essential for effective clinical management of such cases.

## References

[1] Carney JA (1985). Differences between nonfamilial and familial cardiac myxoma. Am J Surg Pathol.

[2] Carney JA, Gordon H, Carpenter PC, Shenoy BV, Go VL (1985). The complex of myxomas, spotty pigmentation, and endocrine overactivity. Med (Baltim).

[3] Stratakis CA, Kirschner LS, Carney JA (2001). Clinical and molecular features of the Carney complex: diagnostic criteria and recommendations for patient evaluation. J Clin Endocrinol Metab.

[4] Kirschner LS, Carney JA, Pack SD, Taymans SE, Giatzakis C, Cho YS (2000). Mutations of the gene encoding the protein kinase A type I-alpha regulatory subunit in patients with the Carney complex. Nat Genet.

[5] Silverman NA (1980). Primary cardiac tumors. Ann Surg.

[6] Bouys L, Bertherat J (2021). Management of endocrine disease: Carney complex: clinical and genetic update 20 years after the identification of the CNC1 (PRKAR1A) gene. Eur J Endocrinol.

[7] Siordia JA. Medical and surgical management of Carney complex. J Card Surg. 2015; 30: 560–7. doi: 10.1111/jocs.12575. [Epub 2015 May 21]. PMID: 25996461 10.1111/jocs.12575 25996461

[8] Hermans K, Jaarsma W, Plokker HW, Cramer MJ, Morshuis WJ (2003). Four cardiac myxomas diagnosed three times in one patient. Eur J Echocardiogr.

[9] Marinakis S, Mircev D, Wauthy P (2013). Cryoablation for a right atrial myxoma arising from the Koch’s triangle: a case report. J Cardiothorac Surg.

[10] Rathore KS, Hussenbocus S, Stuklis R, Edwards J (2008). Novel strategies for recurrent cardiac myxoma. Ann Thorac Surg.

